# TCR and IL-7 Signaling Are Altered in the Absence of Functional GTPase of the Immune Associated Nucleotide Binding Protein 5 (GIMAP5)

**DOI:** 10.1371/journal.pone.0151837

**Published:** 2016-03-29

**Authors:** Xi-Lin Chen, Daniel Serrano, Farnaz Ghobadi, Marian Mayhue, Kasper Hoebe, Subburaj Ilangumaran, Sheela Ramanathan

**Affiliations:** 1 Immunology Division, Department of Pediatrics, Faculty of Medicine and Health Sciences, Université de Sherbrooke, Sherbrooke, J1H 5N4, Québec, Canada; 2 Department of Pediatrics, Division of Cellular and Molecular Immunology, Cincinnati Children's Hospital Medical Center, Cincinnati, OH, 45229, United States of America; 3 Centre de recherche clinique, Université de Sherbrooke, Sherbrooke, J1H 5N4, Québec, Canada; Institut Pasteur, FRANCE

## Abstract

GTPase of the immune associated nucleotide binding protein (GIMAP) family of proteins are expressed essentially in cells of the hematopoietic system. Mutation in the founding member of this gene family, *Gimap5*, results in the lymphopenic phenotype in Bio-Breeding diabetes prone rats. In mice, deletion of functional *Gimap5* gene affects the survival and renewal of hematopoietic stem cells in addition to the defects observed in T cells. Here we show that T cells from OTII TCR-transgenic *Gimap5*^*sph/sph*^ mice do not proliferate in response to its cognate antigen. Furthermore, T cells from *Gimap5* mutant rats and mice show decreased phosphorylation of STAT5 following stimulation with IL-7. Our results suggest that functional *Gimap5* is required for optimal signaling through TCR and IL-7R in T cells.

## Introduction

Even though the GIMAP family of proteins was identified 15 years ago, very little is know about the mechanism of their action [1–3]. In diabetes prone Bio Breeding rats, the *lyp* allele arises from a frame-shift mutation within the GTPase domain of the immune associated nucleotide binding protein 5 (*Gimap5*) gene [4, 5]. Among the proteins of the GIMAP family, *Gimap5* has been studied in detail, as the gene is responsible for the lymphopenic phenotype in the diabetes prone Bio Breeding rats [6]. The domains in GIMAP5 do not show specific homology to any known protein family despite possessing an N-terminal GTPase domain and a C-terminal membrane anchor that localizes the protein to the lysosomes [7].

*Gimap5*^*lyp/lyp*^ rats exhibit a profound T lymphopenia in the secondary lymphoid organs. In rats the *lyp* mutation shortens the lifespan of T cells in the secondary lymphoid organs [8]. T cells from *Gimap5*^*lyp/lyp*^ rats show decreased proliferation following stimulation through TCR/CD3 complex [9]. Similarly, the proliferative response to alloantigens was reduced in amplitude [8]. In normal T cells, upon engagement of the TCR/CD3 complex, activated LCK phosphorylates ZAP70 that is recruited to the CD3 complex. Following phosphorylation of the scaffold protein LAT by activated ZAP70, different signaling modules including phospholipase Cγ(PLCγ) are activated and this culminates in T cell activation [10]. We have shown previously that antibody-mediated cross-linking of the TCR complex results in a comparable pattern of tyrosine phosphorylation in CD4^+^ T cells from *Gimap5*^*lyp/lyp*^ rats [11]. In fact, our findings suggest that the survival defect in GIMAP5 deficient T cells [8, 9, 12] may be related to the impaired calcium (Ca^2+^) response downstream of TCR signaling, that is associated with defects in mitochondrial displacement to buffer the Ca^2+^ influx [11, 13].

Whereas the cell survival defect is confined to T cells in *Gimap5*^*lyp/lyp*^ rats, 2 independently derived lines of *Gimap5*-deficient mice show defects in various hematopoietic cell types including stem cells [14–16]. *Gimap5*^*-/-*^ mice described by Schulteis et al. [14] were generated by deleting exon 2 and part of the exon3 that code for the functional GIMAP5 protein, while *Gimap5*^*sph/sph*^ mice were generated by N-ethyl-Nitrosourea (ENU) mutagenesis [15]. In these mice, a point mutation in the GTPase domain of GIMAP5 destabilizes the protein although the mRNA is expressed normally. Nevertheless, both these lines of mice develop multi-organ failure due to excessive inflammation associated with extra-medullary hematopoiesis. Furthermore, T cells from *Gimap5*^*sph/sph*^ mice do not proliferate in response to CD3/CD28 stimulation [15]. The expression of CD69 is increased with a concomitant down modulation of CD62L expression by T cells from older mice [17]. These changes with age are associated with the loss of the expression of the FOXO family of proteins [17]. However, it is not clear how these alterations are brought about by the loss of functional GIMAP5. In fact, the defects in the Ca^2+^ homeostasis are early events that are observed even before the loss of mitochondrial membrane potential or the increase in apoptosis that is observed later [8, 18]. It is known that the homeostatic survival of naive T cells requires two essential signals, one provided by interleukin-7 (IL-7) and the other by the T cell antigen receptor (TCR) following the interaction with self-peptide:MHC complexes [19]. Therefore we analyzed the proximal signaling events in T cells following stimulation through TCR and IL-7R. Our results suggest that absence of GIMAP5 alters TCR and IL-7 signaling.

## Materials and Methods

### Animals

*Gimap5*^*sph/sph*^ mice have been described previously [15]. *Gimap5*^*sph/sph*^ mice were intercrossed with OT-II TCR transgenic mice to generate OTII *Gimap5*^*sph/sph*^ mice. *Gimap5*^*+/+*^ and *Gimap5*^*lyp/lyp*^ rats in the ACI.1u background have been described before [20]. Mice and rats were housed in micro-isolated sterile cages with unlimited access to food and water under specific pathogen-free conditions. The institutional ethical committee, that follows national standards, approved all experimental protocols. Unless otherwise noted, mice were used at 4 weeks of age and rats between 4 and 6 weeks of age. Mice were sacrificed in 95% CO_2_/5%O_2_ containing chamber after they were anesthetised with isoflurane. ‘Comité Facultaire de Protection des animaux de Université de Sherbrooke’ committee approved all the protocols (protocol number 050-13B).

### Antibodies and reagents

Antibodies against mouse and rat cell surface molecules conjugated to fluorochromes or biotin, and fluorescent streptavidin conjugates were purchased from eBioscience, BD Biosciences or Biolegend (S1 Table). Antibodies against phospho STAT5 (Y694), phospho ZAP70, phospho LAT, STAT5, ZAP70, LAT and FOXO1 were from Cell Signaling Technology or Santa Cruz Biotechnology Inc. Anti-mouse CD3 mAb (2C11) and goat anti-hamster antibodies were from BD Pharmingen Biosciences. Carboxyfluorescein succinimidyl ester (CFSE) was from Invitrogen. RPMI-1640 cell culture medium, fetal bovine serum (FBS), 4G10 that recognizes phospho-tyrosine on tyrosine phosphorylated proteins and anti-alpha-tubulin mAb were from Sigma Aldrich. Nocodazole, colchicine, cytochalasin D and latrunculin B were obtained from Calbiochem. OVA_323-339_ peptide (ISQAVHAAHAEINEAGR) was custom synthesized by Genscript (New Jersey, USA).

### Cell isolation and Flow cytometry

In any given experiment the sex of the mice were matched. OTII mice were males as the OTII transgene is inserted in the Y-chromosome. Mononuclear cell suspensions were prepared from thymus, spleen or pooled lymph nodes (inguinal, axillary, brachial and superficial cervical) and were labeled with indicated antibodies. Murine CD8^+^ SP thymocytes were isolated by depletion of CD4^+^ thymocytes, followed by positive selection (CD4 depletion kit-catalog #11445D and positive selection kit- catalog # 11447D; Dynabeads, Thermoscientific Fisher). Murine CD4^+^ T-cells from pooled lymph nodes were isolated by negative selection (Untouched mouse CD4 kit- catalog # 11415D, Dynabeads, Thermoscientific Fisher). For the purification of T cells from the rats, briefly, lymph node suspensions were incubated with biotinylated anti-rat anti-CD8 antibody (OX8) and anti-Ig kappa antibody (MARK-1) antibody followed by incubation with anti-biotin beads (catalog # 11047-Dynabeads, Thermoscientific Fisher). After purification, the CD8^+^ SP cell purity was >90% and the CD4^+^ T cell purity was >95%. [11, 13, 21]. Expression of cell surface markers was evaluated by flow cytometry using FACS Canto flow cytometer (Becton Dickinson flow cytometry systems) and the data were analyzed using the FlowJo software (Tree Star Inc).

### T cell proliferation assay

CD4^+^ T cells were isolated by negative selection from pooled lymph nodes of control and *Gimap5*^*sph/sph*^ OTII TCR transgenic mice as described in the previous section. Purified CD4^+^ T cell (2×10^4^ cells per well) were stimulated with OVA peptide (0.5 mM) in the presence of 200,000 irradiated splenocytes in 200 μl of RPMI 1640 medium supplemented with penicillin (10^6^U), streptomycin (10^6^U), 2mM L-glutamine, 10mM HEPES buffer, 0.1mM nonessential amino acids, 1 μM pyruvate and 20μM 2-mercaptoethanol and 10% heat-inactivated FCS in 96-well culture plates for 72 h at 37 C in a 5% CO2 humidified atmosphere. 1 μCi of methyl-[^3^H] thymidine (NEN-Life Sciences) was added per well during the last 8 h of culture. Incorporation of radioactivity was measured in a Top Count microplate scintillation counter (PerkinElmer) [21]. In certain experiments, CFSE-labeled lymph node cells were cultured in RPMI 1640 complete medium in the presence of IL-2 (5ng/ml), IL-7 (10ng/ml), IL-15(10ng/ml), PMA/Ionomycin and CD3/CD28. Cells were maintained in culture for 48 and 72 hours, harvested and washed twice with PBS containing 2% FCS. To evaluate the proliferation of T-lymphocyte subsets, total lymphocytes were stained with monoclonal anti-CD3, anti-CD4 and anti-CD8 antibodies and proliferation was determined by CFSE-dye dilution assay as described previously [21].

### T cell stimulation, cell fractionation and immunoblotting

Purified CD4^+^ T cells from pooled lymph nodes were cultured (5 x10^6^ cells/ml) in the presence of 2nM IL-7 or by crosslinking CD3 using anti-CD3 mAb (2C11) followed by goat anti-hamster antibody. CD4^+^ T cells from OT-II TCR transgenic mice were stimulated with OVA_323-339_ (0.5 μM) presented by splenic irradiated APC for the indicated duration. In certain experiments, CD4^+^ T cells were incubated at 37°C in the presence or absence of inhibitors for 30 min and then activated with 2nM IL-7 for 15 min. Cells were then resuspended in ice-cold lysis buffer (10mM PIPES pH 7.30, 10mM NaCl, 3.5mM MgCl_2_, 0.5mM EGTA, 0.5mM EDTA, 1mM DTT) supplemented with protease and phosphatase inhibitors. After incubating in stripping solution (2% SDS, 62.5mM Tris pH 6.8, 100mM 2-mercaptoethanol) for 30 min at 55°C, these blots were blocked and reprobed for total proteins. To obtain cytoplasmic and nuclear fractions, cells were lysed in a hypotonic buffer (10mM HEPES, 10mM KCl, 1.5mM MgCl_2_) containing protease and phosphatase inhibitors. After incubation on ice for 30 min, 0.5% NP-40 was added and the lysates were centrifuged at 400 g for 10 min followed by centrifugation of the supernatant at 16,000 g for 30 min. The supernatant was used as cytoplasmic extract and lysed by 6x sample buffer. Nuclear pellet was resuspended in 1x sample buffer. The purity of the fractions was ascertained by western blot with anti-histone (nuclear) and anti-alpha-tubulin (cytoplasmic) antibodies. Lysates were electrophoresed, transferred onto polyvinylidene difluoride membranes, and processed for immunoblot analysis.

### Statistical analysis

Data were analyzed using two-way ANOVA for statistical comparisons. P values less than 0.05 were considered statistically significant.

## Results

### *Gimap5*^*sph/sph*^ mice exhibit defects in T cell development

**Fig 1:**
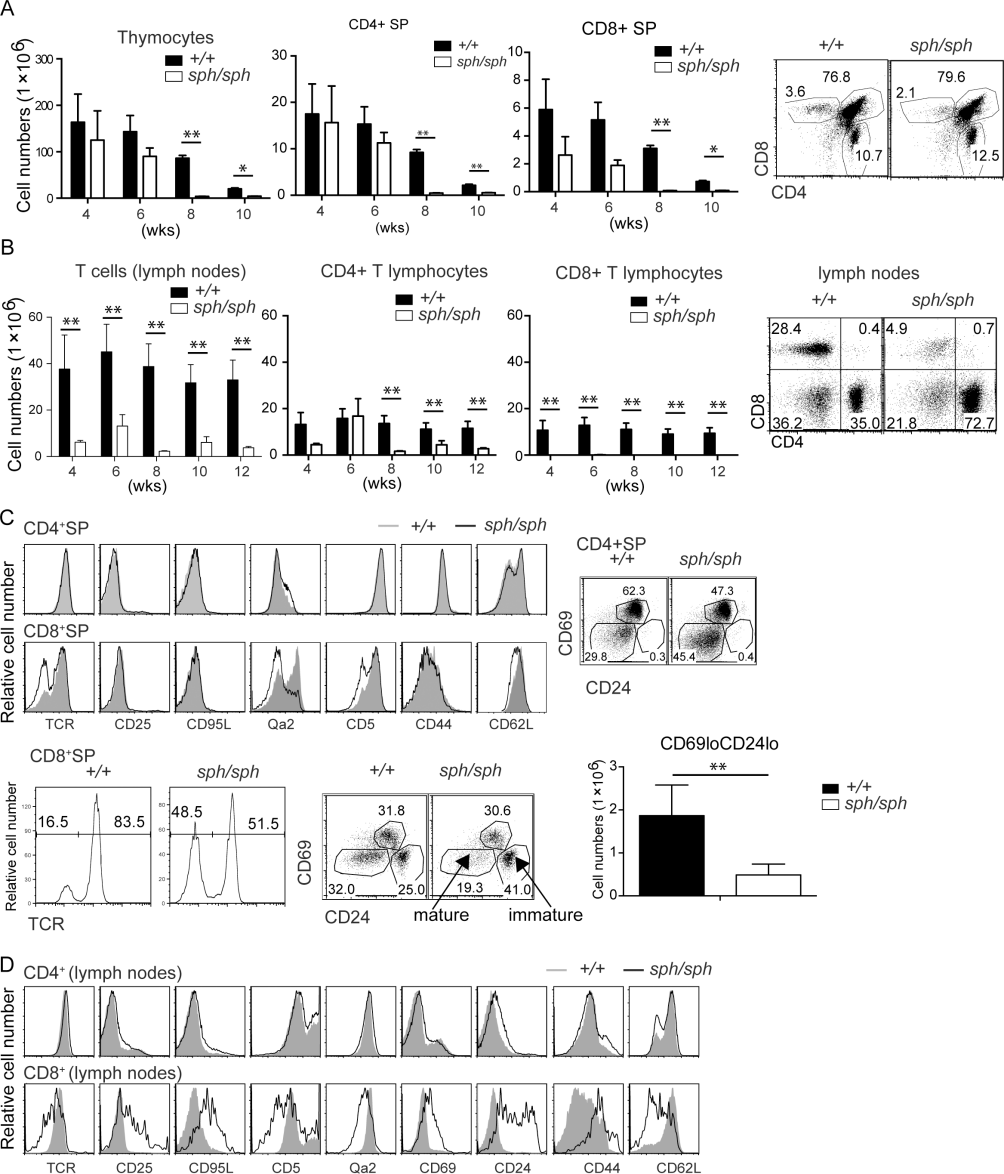
*Gimap5 deficiency* results in disrupted T cell development. (A) Total number of thymocytes from wildtype and Gimap5^*sph/sph*^ mice at different ages (in weeks) was counted by trypan blue staining (bar graphs). Flow cytometric analysis of thymocytes following staining with anti-CD4 and anti-CD8 antibodies in wild-type and *Gimap5*^*sph/sph*^ mice. (B) Total number of T, CD4^+^ and CD8^+^ T cells in pooled lymph nodes (inguinal, axillary, brachial and superficial cervical) from wildtype and Gimap5^*sph/sph*^ mice at different ages (in weeks, from the same groups of mice as in A) were counted by trypan blue staining. The absolute numbers of total T, CD4^+^ and CD8^+^ T cells were calculated by factoring the frequency of CD3^+^ T cells and the frequency of CD4^+^ and CD8^+^ cells within the CD3^+^ gate. (C) The phenotype of CD4^+^ or CD8^+^ SP thymocytes from wild-type and *Gimap5*^*sph/sph*^ mice for the indicated markers is shown as histograms. The data for TCR expression shown in the enlarged histogram and the overlap histogram for CD8^+^SP are from different mice. (D) The phenotype of CD4^+^ or CD8^+^ T cells from wild-type and *Gimap5*^*sph/sph*^ mice for the indicated markers is shown as histograms. Data shown are representative of 3 independent experiments. Each experiment consisted of analyzing 2 individual sex- and age-matched mice from each genotype.

In the rats, the *lyp* mutation manifests itself during the late stages of thymic development [6]. Thus the total numbers of thymocytes are comparable with control rats even though the numbers of T cells show a profound reduction in the secondary lymphoid organs of *Gimap5*^*lyp/lyp*^ rats [6]. Between 4 and 6 weeks of age, there is minimal difference in the total number of thymocytes between control and *Gimap5*^*sph/sph*^ mice, while the thymic cellularity is significantly decreased by 8 weeks of age in *Gimap5*^*sph/sph*^ mice when compared to controls (**Fig 1A**). It is possible that this decrease in thymic cellularity may be secondary to the generalized inflammation that has been reported in other models [22]. There was minimal difference in the proportions of CD4^-^CD8^-^ double negative (DN), CD4^+^CD8^+^ double positive (DP), CD4^+^SP or CD8^+^ SP thymocytes (**Fig 1A**). However, the absolute numbers of T cells were reduced in the periphery of *Gimap5*^*sph/sph*^ mice when compared to control mice (**Fig 1B**). In fact, the lymphopenic phenotype was evident in pooled peripheral lymph nodes (inguinal, brachial, cervical and mesenteric) from 4 weeks of age (**Fig 1B**).

In certain strains of *Gimap5*^*lyp/lyp*^ rats, the proportion of immature thymocytes expressing lower amounts of TCR is increased within the SP CD8^+^ population [18, 23]. As the thymic maturation pathways are better characterized in mice, we analyzed in detail, the phenotype of CD4^+^ and CD8^+^ SP thymocytes in *Gimap5*^*sph/sph*^ mice. In mice, immature SP thymocytes are characterized by CD24^hi^Qa2^lo^TCR^lo^CD69^lo^CD62L^lo^ phenotype, while the CD62L expression is high only on the ‘oldest’ SP thymocytes [24]. The expression of TCR was decreased in the CD8^+^, but not in CD4^+^ SP thymocytes of *Gimap5*^*sph/sph*^ mice when compared to controls (**Fig 1C**). Previous studies have suggested that the maturation of CD8^+^ SP thymocytes was defective as the frequency of CD69^lo^Qa2^lo^ cells was increased in *Gimap5*^*-/-*^ mice [14]. Similarly, the expression of CD24 and CD69 was increased in CD8^+^ SP thymocytes from *Gimap5*^*sph/sph*^ mice [15]. The expression of CD62L is comparable between WT and *Gimap5*^*sph/sph*^ CD4^+^ SP but not in CD8^+^ SP thymocytes from *Gimap5*^*sph/sph*^ mice. Additionally, the increased proportion of TCR low cells in CD8^+^ SP thymocytes (**Fig 1C**), suggests that GIMAP5 can play a role at various stages of T cell development. The frequency of CD24^hi^ CD69^lo^ immature thymocytes is increased in CD8^+^ SP thymocytes, concomitant with the downregulation of TCR, Qa2 and CD5 in CD8^+^ SP thymocytes from *Gimap5*^*sph/sph*^ mice (**Fig 1C**). In contrast, the proportion of late stage mature CD24^lo^CD69^lo^ t thymocytes appears to be increased in the CD4^+^ SP subset (**Fig 1C**). Except for the increase in the frequency of CD24^lo^CD69^lo^ subset, the expression of other markers that define thymocyte maturation is comparable in CD4^+^ SP thymocytes from control and *Gimap5*^*sph/sph*^ mice (**Fig 1C**).

Despite the lymphopenia, in the periphery, the phenotype of CD4^+^ T cells from *Gimap5*^*sph/sph*^ mice is comparable to that of control mice (**Fig 1D**). However, the lymphopenia-induced activation is evident from the increase in the expression of CD44 and decreased expression of CD62L even at 4 weeks of age (**Fig 1D**). In contrast to CD4^+^ T cells, the phenotype of CD8^+^ T cells shows an activated phenotype including upregulation of CD44 and downregulation of CD62L in the lymph nodes of *Gimap5*^*sph/sph*^ mice (**Fig 1D**).

### Signaling through the TCR/CD3 complex is altered in CD4^+^ T cells from *Gimap5*^*sph/sph*^ mice

**Fig 2:**
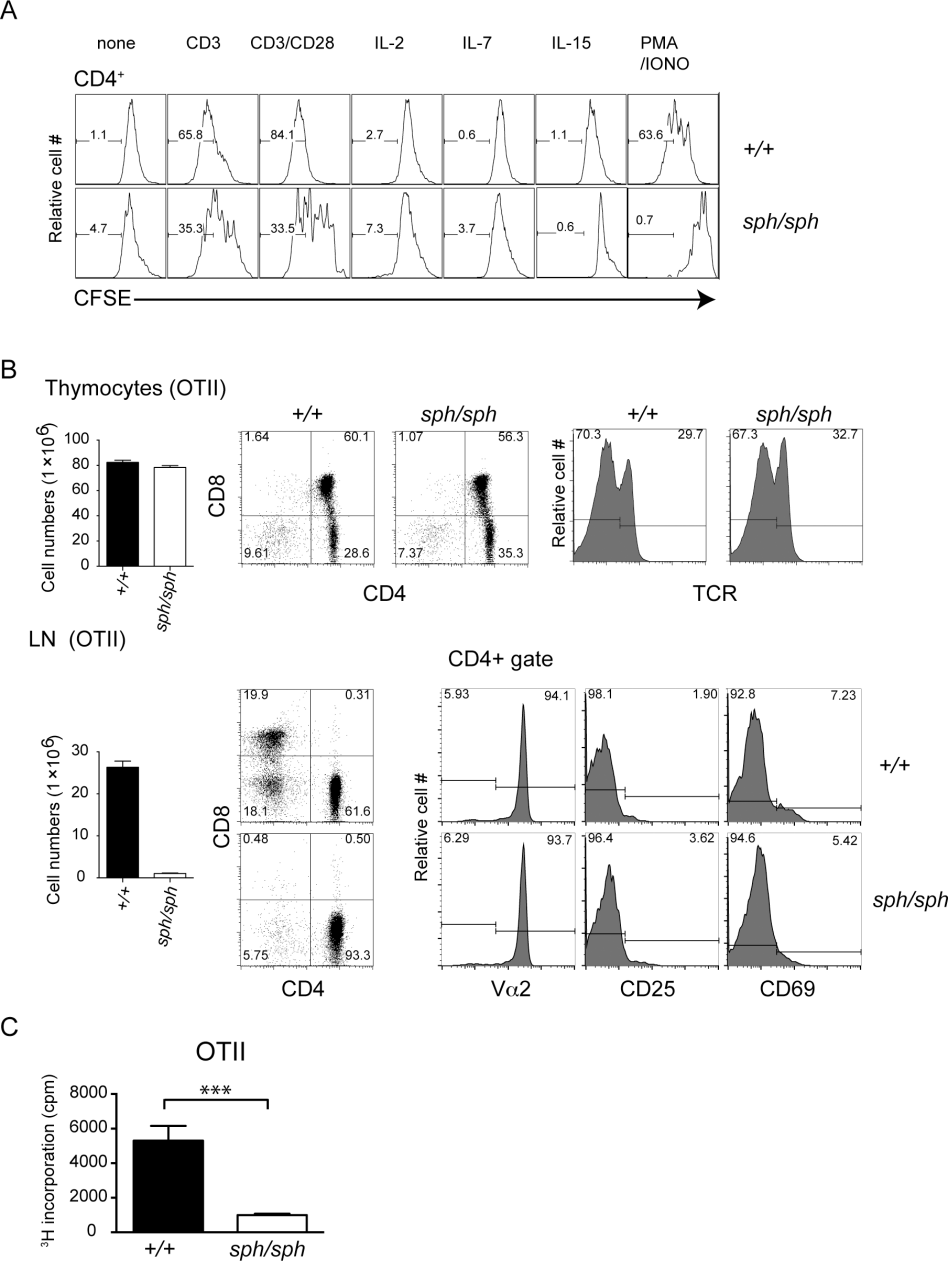
*Gimap5* deficiency results in the absence of proliferative response to cognate antigen in CD4^+^ T cells. (A) CFSE-labeled polyclonal lymphocytes from wildtype and *Gimap5*^*sph/sph*^ mice were cultured with anti-CD3 antibody, anti-CD3&CD28 antibodies, IL-2 (10 ng/ml), IL-7 (5 ng/ml), IL-15 (10 ng/ml) or PMA/Ionomycin for 3 to 5 days. Medium and cytokines were replenished on day 3. The representative histogram shows CFSE dye dilution, indicative of proliferation in the gated CD4^+^ T cells. Cultures stimulated with IL-2, IL-7 and IL-15 were analyzed on day 5. Unstimulated cultures and cultures stimulated with anti-CD3 or anti-CD3/CD28 antibodies and PMA/inomycin were analyzed on day 3. (B) Flow cytometric analysis of thymocytes and lymph node populations by staining with anti-TCR, anti-CD4 and anti-CD8 antibodies in wild-type and *Gimap5*^*sph/sph*^ OTII TCR transgenic mice. Data shown are representative of 3 four-week old mice. (C) Purified CD4^+^ T cells from OT-II TCR-transgenic wild type and *Gimap5*^*sph/sph*^ mice were activated with irradiated APCs in the presence of OVA peptide and cellular proliferation was measured by [^3^H]-thymidine incorporation. Data were pooled from 3 independent mice in each group. *** P value <0.005.

In contrast to T cells from *Gimap5*^*lyp/lyp*^ rats [8, 9], the proliferative response of CD4^+^ T cells from *Gimap5*^*sph/sph*^ mice is greatly reduced following polyclonal stimulation (**Fig 2A**). [15]. To determine if the defect in the proliferative response was also observed in response to nominal antigens, we generated *Gimap5*^*sph/sph*^ mice that expressed the OTII TCR transgene that specifically recognizes the OVA_323-339_ from chicken ovalbumin (**Fig 2B**). Similar to mice with a polyclonal T cell repertoire, the lymphopenic phenotype was evident in the periphery while the thymus did not show any major differences. We did not observe significant differences in the frequency of cells expressing CD25, CD69 or TCRVα2 (OTII transgene) (**Fig 2B**). We purified CD4^+^ T cells from OTII TCR transgenic wild type and *Gimap5*^*sph/sph*^ mice and stimulated them with the cognate peptide presented by irradiated wild type splenocytes. Again, the proliferative response of OTII cells from *Gimap5*^*sph/sph*^ mice was drastically reduced (**Fig 2C**). These observations suggest that GIMAP5 is essential for the antigen-induced proliferative response of mature T cells in mice.

**Fig 3:**
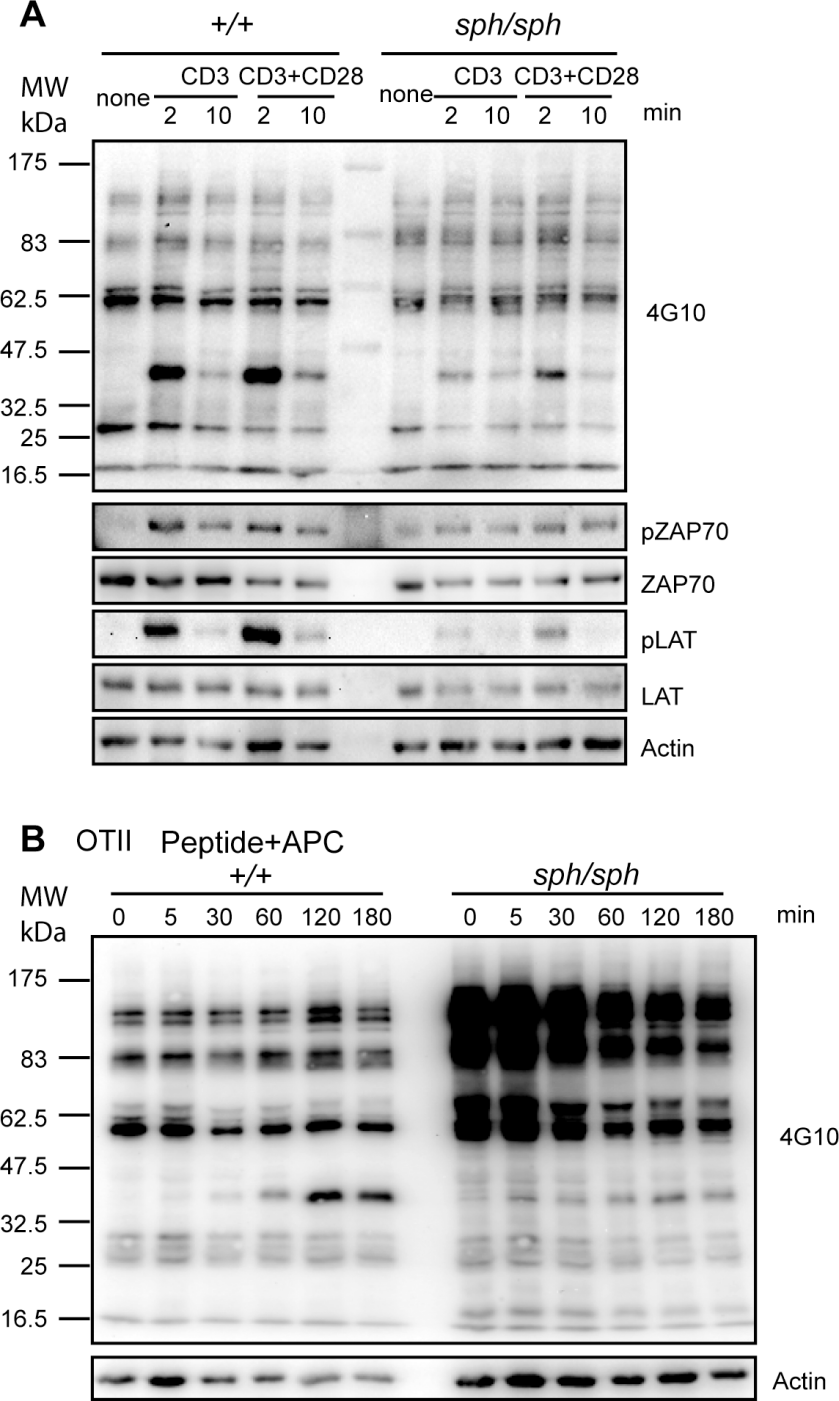
TCR-induced proximal signaling is decreased in *Gimap5* deficient T cells. (A) Purified CD4^+^ T cells from control and *Gimap5*^*sph/sph*^ mice were left un-stimulated or stimulated with 5 μg/μL anti-CD3 or anti-CD3&CD28 antibodies for the indicated duration. Lysates were subjected to Western blot analysis. Representative data from one of 3 independent experiments are shown. (B) CD4^+^ T cells from OT-II TCR-transgenic control and from *Gimap5*^*sph/sph*^ mice were co-cultured with irradiated APC pulsed with OVA peptide at a ratio of APC: CD4^+^ T cells (8:1) for different durations of time. Cell lysates were prepared and phosphorylation of the tyrosine recidue was detected using 4G10 antibody. Representative data from one of 3 independent experiments are shown.

Given that the proliferative response is a late stage event following T cell activation, we assessed TCR-induced proximal signaling in T cells from *Gimap5*^*sph/sph*^ mice. We have shown previously that cross-linking the TCR complex results in comparable pattern of tyrosine phosphorylation in CD4^+^ T cells from *Gimap5*^*lyp/lyp*^ rats [11]. As the proliferation of T cells was dramatically reduced in *Gimap5*^*sph/sph*^ mice, we evaluated the proximal signaling events after cross-linking of CD3 alone or in combination with CD28 using antibodies. There is a global reduction of tyrosine-phosphorylated protein downstream of TCR stimulation in CD4^+^ T cells from *Gimap5*^*sph/sph*^. Data presented in **Fig 3A** also shows that the phosphorylation of ZAP70 was comparable or slightly reduced in CD4^+^ T cells from *Gimap5*^*sph/sph*^ mice, when compared to controls. However, the phosphorylation of LAT was decreased following cross-linking CD3 alone or with CD28 in polyclonal CD4^+^ T cells from *Gimap5*^*sph/sph*^ mice (**Fig 3A**). Due to the limitations in the availability of T cells from OTII TCR transgenic *Gimap5*^*sph/sph*^ mice, we could not carry out an extensive analysis of the proteins involved in T cell signaling pathway. However, we analyzed the pattern of proteins phosphorylated on tyrosine using 4G10 mAb following stimulation with OVA_323-339_ presented by irradiated splenocytes. Data presented in **Fig 3B** shows reduced phosphorylation of a protein between 32 and 47kDa (similar to **Fig 3A**) in T cells from OTII TCR transgenic *Gimap5*^*sph/sph*^ mice, even though the intensity of total phosphorylation appears to be increased. These observations suggest that the generation of proximal signals following TCR stimulation requires the presence of GIMAP5.

### IL-7–induced STAT5 phosphorylation is decreased in CD4^+^ T cells from *Gimap5*^*sph/sph*^ mice

**Fig 4:**
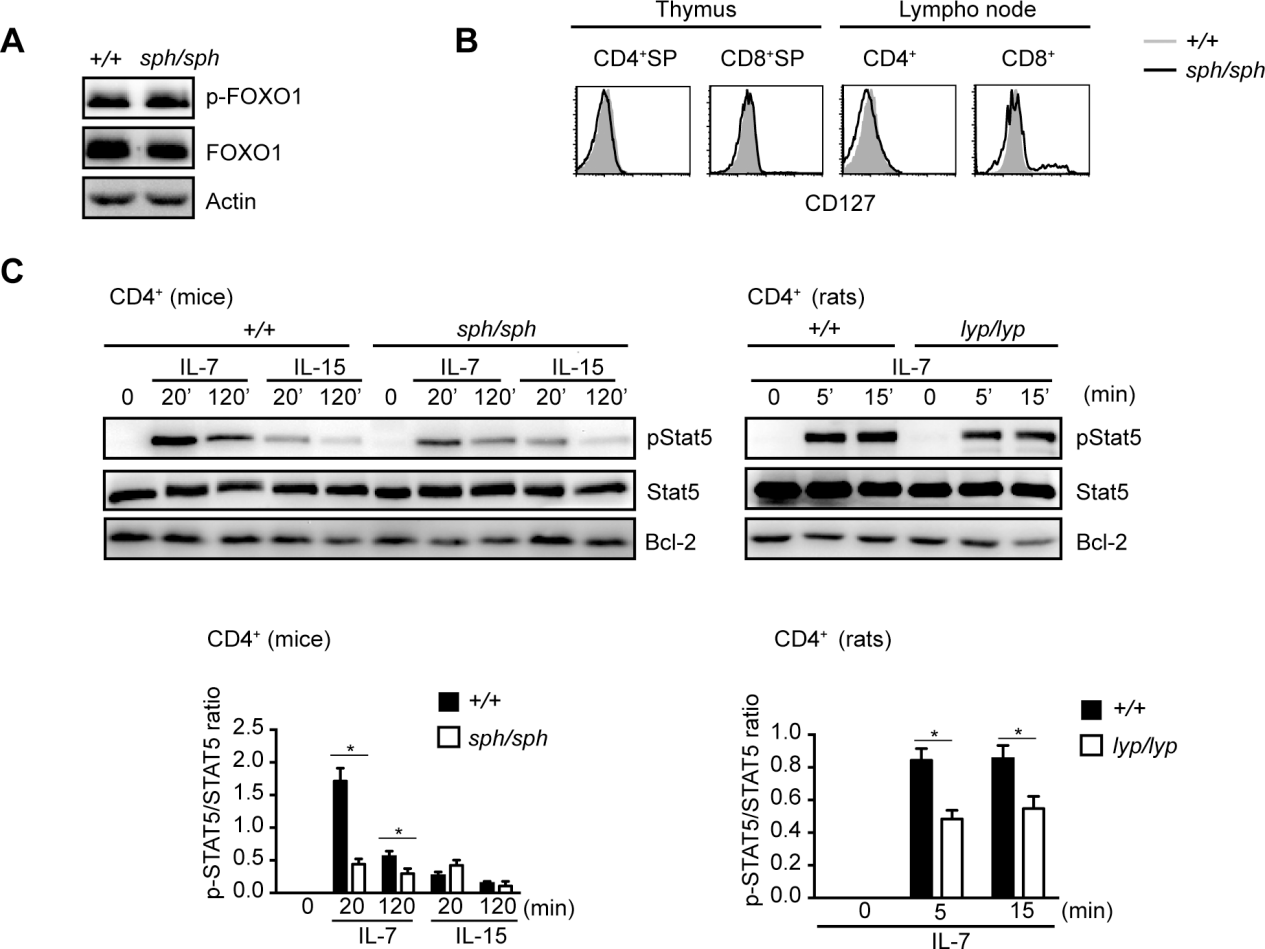
IL-7-induced STAT5 phosphorylation is down regulated in *Gimap5*-deficient T cells. (A) CD4^+^ T cells were purified from freshly isolated lymphocytes from wild type and *Gimap5*^*sph/sph*^ mice. Equal amounts of protein lysates were subjected to Western blot analysis for the expression of FOXO1 and pFOXO1. Data shown are representative of 3 independent experiments. (B) Thymocytes and lymph node cells were stained with antibodies to CD4 and CD8. CD127 expression was assessed in gated CD4^+^ SP or CD8^+^ SP thymocytes and CD4^+^ or CD8^+^ T cell subsets by flow cytometry. Data shown are representative of 3 independent experiments. (C) Purified CD4^+^ T from *Gimap5*^*sph/sph*^ mice or *Gimap5*^*lyp/lyp*^ rats were stimulated with IL-7 or IL-15 at 37°C. At the indicated time points the cells were lysed and subjected to Western blot analysis for the detection of phosphorylated STAT5, total STAT5 or Bcl-2. Lower panel represents the densitometric data pooled from 3 independent experiments. * p value < 0.05.

In addition to signals through the TCR complex, IL-7 mediated signals are required for the survival of T lymphocytes in the periphery. Previous reports have shown that the expression of IL-7 receptor α chain (CD127) is decreased in T cells from *Gimap5*^*sph/sph*^ mice [15] which correlated with the loss of FOXO family of proteins that regulate its expression [17]. Since the loss of the FOXO proteins increased with age, we confirmed the presence of FOXO1 protein at 4 weeks of age, the time point when most of the experiments were carried out. At 4 weeks of age, the expression of FOXO1 protein and its phosphorylation status in CD4^+^ T cells from *Gimap5*^*sph/sph*^ mice were comparable to their counterparts from control mice (**Fig 4A**) [17]. As the expression of CD127 was also comparable in T cells from control and mutant mice in SP thymocytes but showed slight reduction in peripheral CD4^+^ T cells at 4 weeks of age (**Fig 4B**), we determined whether IL-7 mediated signals are altered in T cells from *Gimap5*^*sph/sph*^ mice. As shown in **Fig 4C**, phosphorylation of STAT5 was decreased in CD4^+^ T cells from *Gimap5*^*sph/sph*^ mice when compared to controls. The reduction was specific to signals through the IL-7 receptor, as IL-15-induced phosphorylation of STAT5 was comparable between the 2 groups (**Fig 4C**, middle panel). The reduced IL-7-induced phosphorylation of STAT5 was also observed in CD4^+^ T cells from *Gimap5*^*lyp/lyp*^ rats (**Fig 4C**, right panel). Thus, GIMAP5 deficiency affects signaling through the IL-7 receptor in addition to the signals through the TCR/CD3 complex.

### Nuclear translocation of pSTAT5 is not dependent on microtubules in rodent T cells

**Fig 5:**
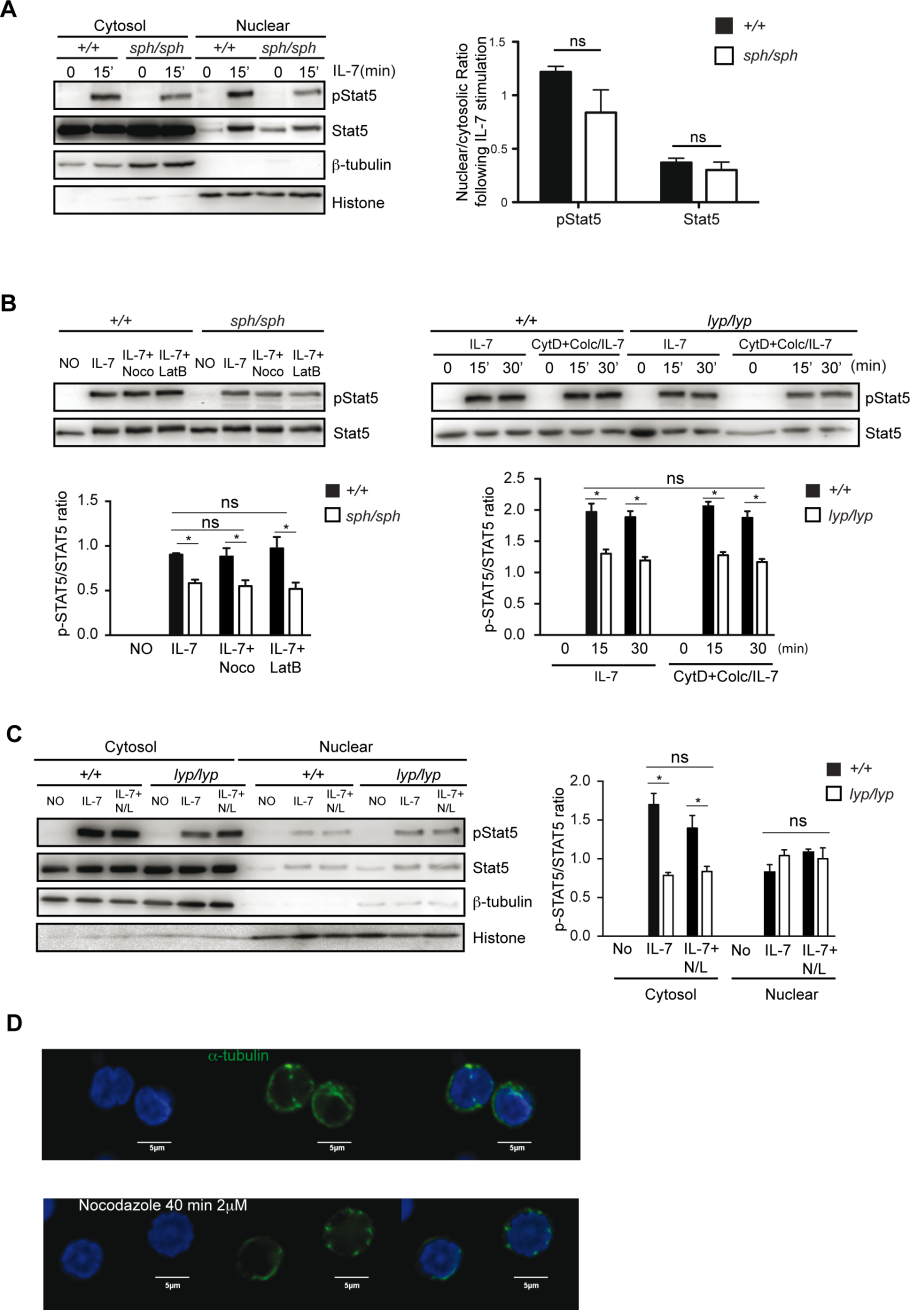
Nuclear translocation of pSTAT5 is not dependent on microtubules in murine T cells. Purified CD4^+^ T cells from *Gimap5*^*sph/sph*^ mice (A, B) or *Gimap5*^*lyp/lyp*^ rats (B, C) were cultured with IL-7 in the absence or presence of cytoskeleton inhibitors nocodazole (Noco, 2 μM) or latrunculin B (LatB, 15 μg/ml) or both together (N/L in C), Cytochalasin D (CytD, 20 μM) or/and Colchicine (Colc, 10 μM) for 15 min. Western blot analysis of whole-cell lysate (B upper two panels), cytosolic and nuclear fractions (A, C) was performed using anti-phospho-STAT5 and anti-STAT5 antibodies. Antibodies to alpha-tubulin and histone were used as markers for cytosolic and nuclear fractions, respectively. The data presented are representative of 3 independent experiments. Bar histogram represent the densitometric data pooled from 3 independent experiments. * p value < 0.05; ns = not significant. D) Confocal image of naïve CD4^+^ T cells from rats which were treated (or not) with Nocadazole (2 μM; to disrupt microtubules) for 40 min and stained with anti-alpha-tubulin (counterstained with DAPI for the nucleus) to visualize the disruption of microtubules.

Phosphorylated STAT5 tetramerizes and is transported to the nucleus along microtubules [25]. As we had observed that the movement of mitochondria on microtubules for buffering Ca^2+^ was defective in T cells from *Gimap5*^*lyp/lyp*^ rats [11], we hypothesized that the movement of pSTAT5 to the nucleus would also be reduced in T cells from *Gimap5*^*sph/sph*^ mice following IL-7 stimulation. Therefore we analyzed the ratio between cytosolic and nuclear phospho STAT5 (pSTAT5) in cell lysates following stimulation with IL-7. While the total amount of pSTAT5 was decreased in both the cytosol and nuclear fractions (**Fig 5A, left**), to our surprise, we did not observe any difference in the relative amounts of pSTAT5 that was present in the nucleus (**Fig 5A**) in lysates from *Gimap5*^*sph/sph*^ mice. These observations suggested that the proportion of pSTAT5 that is translocated to the nucleus was comparable in T cells from control and GIMAP5 deficient mice. In human T cells, IL-7-induced phosphorylation of STAT5 as well as its translocation to the nucleus was shown to be dependent on cytoskeleton using specific inhibitors [25]. However, pretreatment of resting CD4^+^ T cells with different inhibitors of actin polymerization (cytochalasin D and lanturnculin B) or microtubule polymerization (nocodazole and colchicine) had minimal influence on the level of phosphorylation of STAT5 following IL-7 stimulation in CD4^+^ T cells either from mice or rats (**Fig 5B and 5C**). We have shown previously that 2mM nocadazole disrupted the microtubular network (**Fig 5D)**, as it disrupted the movement of mitochondria on microtubules [11]. These inhibitors had minimal influence on the translocation of pSTAT5 to the nucleus (**Fig 5C**). As the movement of pSTAT5 to the nucleus appears not to be dependent upon the microtubule network in T cells from rats and mice, it is not unexpected that we did not observe any effect of the loss of GIMAP5 on the movement of pSTAT5.

## Discussion

The effect of mutations in *Gimap5* begins to be manifested from SP stage of T cell development, even though *Gimap5* expression is detected in DP thymocytes [17]. The development of CD8^+^ T cells is affected more severely than the CD4^+^ T cells as a consequence of the mutations in *Gimap5* both in rats and in mice [18, 23]. Based on the phenotype of thymocytes and peripheral T cells in HY TCR transgenic mice, it is clear that GIMAP5 does not affect negative selection [17]. However the survival of positively selected HY-TCR [17] or OT-1 TCR (our unpublished observations) was severely compromised in the absence of GIMAP5. Even though lymphopenia is evident in the secondary lymphoid organs, the phenotype of CD4^+^ T cells at 4 weeks of age is not dramatically altered. On the other hand, the immature phenotype of the CD8^+^ T cells persists in the secondary lymphoid organs of *Gimap5*^*sph/sph*^ mice. As the mutant T cells are lost from the periphery following reconstitution of normal T cells [17], it is difficult to distinguish the effect of lymphopenia-induced proliferation from that of T cell maturation defects.

In contrast to what is observed in the rats, *Gimap5* mutation affects the proliferation of T cells in mice [9, 15]. While the proximal signaling events following the engagement of the CD3/TCR complex appear to be normal in T cells from *Gimap5*^*lyp/lyp*^ rats [11], the amplitude of these signals are reduced in T cells from *Gimap5*^*sph/sph*^ mice. Furthermore, in contrast to the rats, the mutant T cells in mice do not proliferate in response to polyclonal stimulation [17] or cognate antigen (**Fig 2**). Thus, it is not clear at what stage the T cell signaling process is aborted. However, it is interesting that these T cells differentiate into Th17 cells more readily in the absence of GIMAP5 [17]. This skewing towards Th17 phenotype may be the consequence of the constitutive phosphorylation of mTORC1 pathway [26]. As the proliferative defect in the mutant mice encompasses different hematopoietic subsets, it is possible that the species-specific functions of GIMAP5 regulate the severity of the phenotype. Also it is possible that in mice the functions of *Gimap5* are modulated by *Gimap3*, but not in rats and humans, where *Gimap3* is a pseudogene [27].

On the other hand, the function of GIMAP5 appears to be the same in IL-7 mediated signaling pathway. Unlike AKT that is constitutively phosphorylated in *Gimap5* mutant T cells [26], STAT5 phosphorylation is dependent on signals through the IL-15 or IL-7 receptors (Fig 4). Aksoylar et al. have shown that the expression of IL-7R decreases with age due to down regulation of FOXO1 proteins [17]. At 4 weeks of age the expression of FOXO1, its phosphorylation status and the cell surface expression of IL-7R alpha subunit (CD127) was reduced in T cells from *Gimap5*^*sph/sph*^ mice. Thus, we cannot rule some subtle functional defects in the IL-7R signaling machinery. The decreased phosphorylation of STAT5 following stimulation with IL-7 points to defects at membrane proximal steps. Disruption of lipid rafts can alter IL-7-induced JAK3 phosphorylation [25]. Recently actin cytoskeleton has been shown to be required for endocytosis of IL-4 receptor, enrichment of the receptor dimers in the endosomes and JAK3 phosphorylation in HEK 293T cells [28]. It is also not clear if the signaling defect in IL-7 is related to the defects associated with the movement of the mitochondria on microtubules that we had reported previously [11]. In human T cells, IL-7-mediated STAT5 phosphorylation and translocation of pSTAT5 to the nucleus has been shown to be dependent on microtubules [25]. The data presented in **Fig 5**, suggest that translocation of pSTAT5 to the nucleus may not be dependent on the microtubules in mice and rats. Therefore it is not surprising that the proportion of pSTAT5 in the nucleus was comparable in T cells from control and *Gimap5*^*sph/sph*^ mice. Also, it is possible that the decrease in IL-7 induced phosphorylation of STAT5 is a consequence of the lymphopenic environment. The expression of the IL-7R has been shown to decreased in the lymphopenic environment to maintain the diversity of the T cell repertoire [29]. Dissecting the relative contribution of lymphopenia to the signaling pathways in naïve T cells where GIMAP5 appears to be required for both TCR and IL-7 mediate signaling, will be a challenge.

## Supporting Information

S1 Table List of antibodies used in this study. (DOCX)

## Abbreviations

Ca^2+^: calcium
DN: double negative
DP: double positive
GIMAP5: GTPase of the immune associated nucleotide binding protein 5
IP_3_: inositol triphosphate
PLCγ: phospholipase Cγ
ENU: N-ethyl-Nitrosourea
SP: single positive
TCR: T cell antigen receptor
TG: thapsigargin
