# Supplementary material for: TCR and IL-7 Signaling Are Altered in the Absence of Functional GTPase of the Immune Associated Nucleotide Binding Protein 5 (GIMAP5)
Source: PLoS One. 2016 Mar 29;11(3):e0151837. doi: 10.1371/journal.pone.0151837 (PMC4811415; doi:10.1371/journal.pone.0151837)
Supplement: S1 Table — (DOCX) [file pone.0151837.s001.docx]

**S1 Table. List of antibodies used in this study**

| **Clone** | **Antibodies** | **#cat.** | **Company** |
| --- | --- | --- | --- |
| 145-2C11 | CD3ε-Alexa Fluor® 488 | 100321 | Biolegend |
| 145-2C11 | CD3e-PE-Cy5 | 15-0031-82 | eBioscience |
| GK1.5 | CD4-PE | 12-0041-85 | eBioscience |
| GK1-5 | CD4-APC | 17-0041-81 | eBioscience |
| GK1.5 | CD4-APC-Cy7 | 100414 | Biolegend |
| 53-6.7 | CD8α-PE | 12-0081-85 | eBioscience |
| 53-6.7 | CD8α-PE-Cy7 | 25-0081-82 | eBioscience |
| 53-6.7 | CD8α-APC | 17-0081-83 | eBioscience |
| 53-6.7 | CD8α-APC-Cy7 | 557654 | BD Biosciences |
| A7R34 | CD127-Alexa Fluor® 488 | 135018 | Biolegend |
| H57-597 | TCRβ-FITC | 11-5961-82 | eBioscience |
| 3C7 | CD25-APC | 101910 | Biolegend |
| 53-7.3 | CD5-PE | 553022 | BD Biosciences |
| MFL3 | CD95L-Biotin | 13-5911 | eBioscience |
| IM7 | CD44-PE-Cy7 | 25-0441-82 | eBioscience |
| MEL-14 | CD62L-APC | 17-0621-83 | eBioscience |
| H1.2F3 | CD69-PECy7 | 25-0691-82 | eBioscience |
| M1/69 | CD24-FITC | 553261 | BD Biosciences |
| B20.1 | TCRvalpha2 | 11-5812-81 | eBioscience |
|  | APC Streptavidin | 554067 | BD Biosciences |
| 69H1-9-9 | Qa2-FITC | 11-5996 | eBioscience |
